# Reduced Necrosis and Content of Apoptotic M1 Macrophages in Advanced Atherosclerotic Plaques of Mice With Macrophage-Specific Loss of Trpc3

**DOI:** 10.1038/srep42526

**Published:** 2017-02-10

**Authors:** Sumeet Solanki, Prabhatchandra R. Dube, Lutz Birnbaumer, Guillermo Vazquez

**Affiliations:** 1 Department of Physiology and Pharmacology, and Center for Hypertension and Personalized Medicine, University of Toledo College of Medicine and Life Sciences, University of Toledo Health Science Campus, 3000 Transverse Dr., Toledo, Ohio 43614, USA; 2 Neurobiology Laboratory, National Institute of Environmental Health Sciences, 111 TW Alexander Dr., Research Triangle Park, North Carolina 27709, USA; 3 Institute of Biomedical Research (BIOMED UCA-CONICET), Faculty of Medical Sciences, Av. Alicia Moreau de Justo 1600, C1107AFF, Buenos, Aires, Argentina

## Abstract

In previous work we reported that ApoeKO mice transplanted with bone marrow cells deficient in the Transient Receptor Potential Canonical 3 (TRPC3) channel have reduced necrosis and number of apoptotic macrophages in advanced atherosclerotic plaques. Also, *in vitro* studies with polarized macrophages derived from mice with macrophage-specific loss of TRPC3 showed that M1, but not M2 macrophages, deficient in Trpc3 are less susceptible to ER stress-induced apoptosis than Trpc3 expressing cells. The questions remained (a) whether the plaque phenotype in transplanted mice resulted from a genuine effect of Trpc3 on macrophages, and (b) whether the reduced necrosis and macrophage apoptosis in plaques of these mice was a manifestation of the selective effect of TRPC3 on apoptosis of M1 macrophages previously observed *in vitro*. Here, we addressed these questions using Ldlr knockout (Ldlr^−/−^) mice with macrophage-specific loss of Trpc3 (MacTrpc3^−/−^/Ldlr^−/−^ → Ldlr^−/−^). Compared to controls, we observed decreased plaque necrosis and number of apoptotic macrophages in MacTrpc3^−/−^/Ldlr^−/−^ → Ldlr^−/−^ mice. Immunohistochemical analysis revealed a reduction in apoptotic M1, but not apoptotic M2 macrophages. These findings confirm an effect of TRPC3 on plaque necrosis and support the notion that this is likely a reflection of the reduced susceptibility of Trpc3-deficient M1 macrophages to apoptosis.

Atherosclerosis is the main cause of coronary heart disease, a leading determinant of morbidity and mortality in the United States^1^. Clinical manifestations of atherosclerosis include ischemic symptoms due to plaques causing critical stenosis or thromboembolic events that often follow the rupture of vulnerable plaques^2^,^3^. The process that mediates atherosclerotic plaque formation and growth involves an intricate interaction between smooth muscle cells, endothelial cells, monocyte-derived macrophages, and a myriad of cytokines and inflammatory mediators that lodge in the plaque^4^. Among these, macrophages are recognized as central players throughout all stages of the disease. Macrophage apoptosis is one of the most influential events in shaping plaque features and determining their progression. In advanced plaques in particular, where the removal of apoptotic macrophages –efferocytosis- is impaired, the accumulation of apoptotic bodies favors the growth of the necrotic core, mostly through secondary necrosis, and adds to the factors that contribute to plaque instability^5^,^6^. An often ignored aspect is the fact that the macrophage population in the plaque is composed of phenotypically and functionally distinct subsets. The M1 or inflammatory, and M2 or anti-inflammatory types dominate in atherosclerosis^7^,^8^,^9^. The relative abundance of M1 and M2 macrophages varies with lesion stage, generally increasing as lesion progresses. Yet, both types co-exist throughout lesion development, intermediate phenotypes seem to exist and phenotype inter-conversion can occur^8^,^10^.

Transient Receptor Potential Canonical 3 (TRPC3) is a calcium-permeable channel that belongs to the TRPC family of non-selective cation channels^11^. Under physiological conditions TRPC3 is activated by receptor-dependent activation of phosphoinositide-specific phospholipases and also exhibits significant constitutive function^11^,^12^. The participation of TRPC3 in molecular and cellular processes associated to the pathophysiology of cardiovascular disease is well documented^13^,^14^,^15^. Our laboratory was the first in examining the functions of TRPC3 in macrophage biology and its implications in atherosclerosis^4^. One of these studies showed that loss of TRPC3 function impairs the survival of naïve bone marrow-derived macrophages^16^, leading to the speculation that in the setting of atherosclerosis, deletion of macrophage Trpc3 would lead to accumulation of apoptotic cells and increased plaque necrosis. Contrary to this prediction, our recent observations in Apoe knockout mice transplanted with bone marrow from Trpc3^−/−^/Apoe^−/−^ or Trpc3^+/+^/Apoe^−/−^ animals, showed that some of the typical features of advanced plaques were actually alleviated by bone marrow deletion of Trpc3^17^. Compared to control animals, advanced lesions in the aortic root of mice with bone marrow deletion of Trpc3 showed reduction in necrosis and in the content of apoptotic macrophages, with no changes in lesion size or cellularity^17^. This was interpreted as potentially reflecting an effect of TRPC3 on polarized macrophages, which are abundant in plaques, and different from the observed *in vitro* effect on naïve macrophages. In line with this interpretation, M1 but not M2 macrophages prepared from mice with Trpc3 deficient bone marrow or from mice with macrophage-specific loss of TRPC3, were less sensitive to endoplasmic reticulum (ER) stress-induced apoptosis than TRPC3 expressing M1 cells^17^,^18^. Among the immune cells found in murine atherosclerotic plaques, lymphocytes, neutrophils and dendritic cells do not express TRPC3^19^,^20^,^21^,^22^,^23^. Thus, despite the fact that the bone marrow cells used in the studies described above were derived from mice with global deletion of Trpc3, it was reasonable to conclude that the observed plaque phenotype was presumably due to the impact of Trpc3 deficiency on macrophage apoptosis^17^. However, two important questions remained unanswered. First, whether Trpc3 deletion in bone marrow cells other than myeloid precursors – v.g., bone marrow-derived progenitors for endothelial and smooth muscle cells- could have contributed to the plaque phenotype in mice with bone marrow deletion of Trpc3. Second, whether the reduced necrosis and macrophage apoptosis observed in advanced plaques of the transplanted animals was to some extent a consequence of the selective effect of TRPC3 on apoptosis of M1 macrophages previously observed *in vitro*^17^,^18^.

To address these important questions, in the present work we used the low density lipoprotein receptor knockout (Ldlr^−/−^) mouse model of atherosclerosis transplanted with bone marrow from mice with macrophage-specific loss of TRPC3 (MacTrpc3^−/−^/Ldlr^−/−^) or control Ldlr^−/−^ animals and examined the characteristics of advanced atherosclerotic lesions (14 weeks on high fat diet). In agreement with previous findings, we observed a marked decrease in necrosis and number of apoptotic macrophages in Ldlr^−/−^ mice with macrophage-specific loss of TRPC3 compared to controls. Notably, immunohistochemical examination of advanced plaques revealed a reduction in apoptotic M1 macrophages, but not in apoptotic M2 cells. These observations support the previous *in vitro* findings on polarized macrophages, and indicate that in advanced atherosclerotic plaques the beneficial impact of macrophage-specific deletion of Trpc3 likely reflects the reduced susceptibility of Trpc3-deficient M1 macrophages to apoptosis in the plaque setting.

## Results

In this study we directly examined the impact of macrophage-specific deletion of Trpc3 on the characteristics of advanced atherosclerotic lesions in Ldlr^−/−^ mice fed a conventional high fat diet. We undertook a bone marrow transplantation approach in which Ldlr^−/−^ mice were used as recipients of bone marrow from mice with macrophage-specific loss of TRPC3 (on Ldlr^−/−^ background, MacTrpc3^−/−^/Ldlr^−/−^). Six week-old female Ldlr^−/−^ mice were lethally irradiated and transplanted with bone marrow from Ldlr^−/−^ (control group: Ldlr^−/−^ → Ldlr^−/−^) or from MacTrpc3^−/−^/Ldlr^−/−^ mice (study group: MacTrpc3^−/−^/Ldlr^−/−^ → Ldlr^−/−^) following protocols previously described^17^,^24^. Conversion to the donor’s phenotype was confirmed four weeks after transplantation by PCR on gDNA from peripheral blood (Supplemental Figure S1). Next, all mice were placed on a high fat diet (HFD; TD.88137, Harlan Teklad) for 14 weeks. We previously showed that bone marrow-transplanted Ldlr^−/−^ mice subjected to this regimen develop advanced atherosclerotic lesions^24^. At the end of the diet period, bone marrow-derived macrophages were prepared from both groups of mice and the levels of Trpc3 mRNA were examined by qRT-PCR. As expected, there was a marked decrease in TRPC3 expression in macrophages derived from mice with macrophage-specific loss of TRPC3, but not in control cells (Supplemental Figure S2).

**Figure 1:**
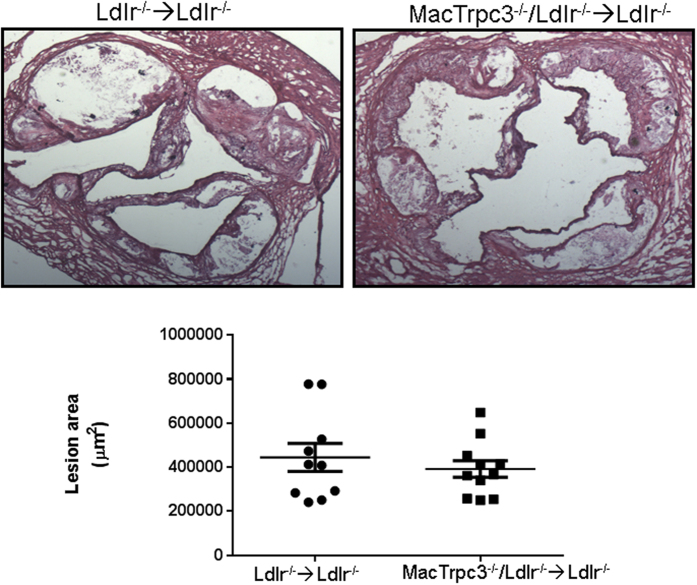
Aortic root sections from Ldlr^−/−^ → Ldlr^−/−^ or MacTrpc3^−/−^/Ldlr^−/−^ → Ldlr^−/−^ mice that were maintained on a high fat diet for 14 weeks were stained with hematoxylin-eosin to evaluate atherosclerotic plaque area. Quantitations of mean stained areas are shown (mean ± SEM). Ldlr^−/−^ → Ldlr^−/−^ (n = 10), MacTrpc3^−/−^/Ldlr^−/−^ → Ldlr^−/−^ (n = 11). Total magnification: 50x.

**Figure 2:**
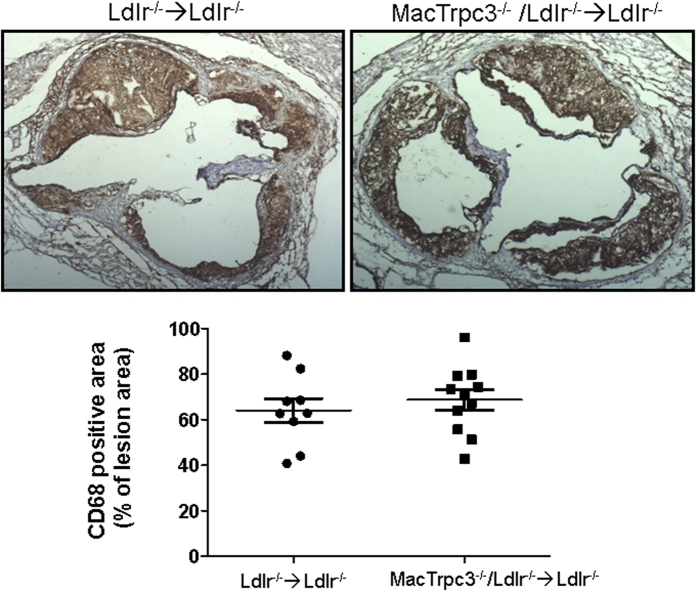
Aortic root sections from Ldlr^−/−^ → Ldlr^−/−^ or MacTrpc3^−/−^/Ldlr^−/−^ → Ldlr^−/−^ mice that were maintained on a high fat diet for 14 weeks, were stained with anti-CD68 antibody to evaluate macrophage content. Shown are representative sections of CD68 staining and corresponding quantitation of the stained areas (mean ± SEM) from Ldlr^−/−^ → Ldlr^−/−^ (n = 9) or MacTrpc3^−/−^/Ldlr^−/−^ → Ldlr^−/−^ (11) mice. Total magnification: 50x.

At sacrifice, body weight, total cholesterol and triglycerides were recorded. As shown in Table S1, these parameters were comparable between both groups of mice. Cholesterol distribution in lipoprotein fractions was also similar between both groups (not shown). To examine the impact of macrophage-specific loss of TRPC3 on typical features of advanced atherosclerotic lesions we performed histological analysis of aortic root sections as in^17^,^25^. Based on the morphometric evaluation of hematoxylin-eosin stained sections, we found no significant differences in total plaque area between the control and study groups [444,777 ± 63,171 μm^2^ (n = 10) vs. 392,003 ± 37,823 μm^2^ (n = 11), for Ldlr^−/−^ → Ldlr^−/−^ and MacTrpc3^−/−^/Ldlr^−/−^ → Ldlr^−/−^ mice, respectively, p = 0.47; Fig. 1]. Neutral lipid content, assessed by Oil Red O (ORO) staining, was not different [365,752 ± 44,742 μm^2^ (n = 10) vs. 377,134 ± 28,600 μm^2^ (n = 11), for Ldlr^−/−^ → Ldlr^−/−^ and MacTrpc3^−/−^/Ldlr^−/−^ → Ldlr^−/−^ mice, respectively, p = 0.82; Supplemental Figure S3]. The macrophage content of aortic root plaques was also similar between groups [CD68-positive area normalized by total lesion area: 64.09 ± 5.18% (n = 9) vs. 68.67 ± 4.49% (n = 11), for Ldlr^−/−^ → Ldlr^−/−^ and MacTrpc3^−/−^/Ldlr^−/−^ → Ldlr^−/−^ mice, respectively, p = 0.51; Fig. 2].

**Figure 3:**
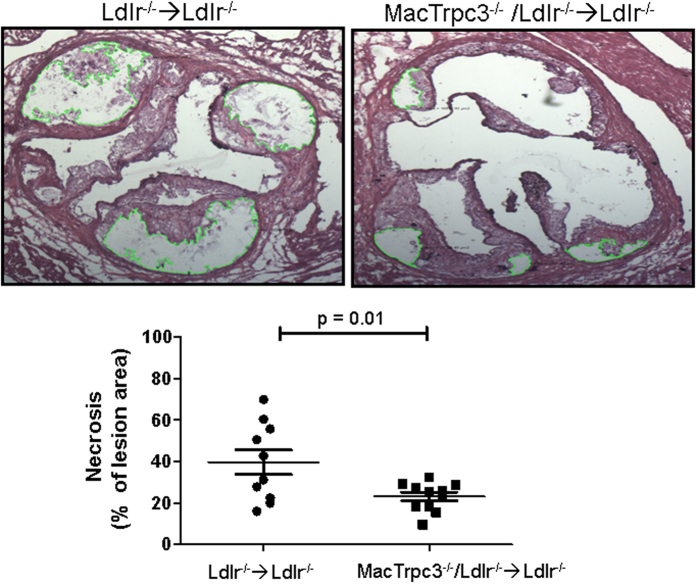
Aortic root sections from Ldlr^−/−^ → Ldlr^−/−^ or MacTrpc3^−/−^/Ldlr^−/−^ → Ldlr^−/−^ mice fed a high fat diet during 14 weeks were stained with hematoxylin and eosin to evaluate necrosis. Shown are representative sections with areas of necrosis delimited by green traces. Quantification of necrotic areas is provided as the percent of necrosis within the total lesion area (mean ± SEM). Total magnification: 50x.

In most advanced plaques that develop after 14 weeks of HFD feeding, the presence of areas of necrosis is clearly noticeable. When we compared the necrosis content of plaques between MacTrpc3^−/−^/Ldlr^−/−^ → Ldlr^−/−^ and Ldlr^−/−^ → Ldlr^−/−^ mice, we observed a significant reduction in percent necrotic area in plaques from mice with macrophage-specific loss of TRPC3 compared to control animals [respectively, 23.12 ± 2.07% (n = 11) vs. 39.63 ± 5.93% (n = 10), p = 0.01); Fig. 3]. Cap thickness was similar between both groups [14.5 ± 1.2 vs. 14.3 ± 0.9 μm, n = 9, p = 0.907]. Based on Movat’s pentachrome staining, the collagen content in aortic root sections from MacTrpc3^−/−^/Ldlr^−/−^ → Ldlr^−/−^ was not different from that in Ldlr^−/−^ → Ldlr^−/−^ mice [respectively, 7.78 ± 0.92% (n = 8) vs. 7.06 ± 0.97% (n = 9), p = 0.602; Supplemental Figure S4]. In addition, immunostaining for α-smooth muscle actin did not show differences in smooth muscle content in the aortic root sections between the two groups of animals [9.60 ± 2.0% (n = 6) vs. 8.8 ± 1.6% (n = 6), for Ldlr^−/−^ → Ldlr^−/−^ and MacTrpc3^−/−^/Ldlr^−/−^ → Ldlr^−/−^ mice, respectively, p = 0.807; Supplemental Figure S4].

Macrophage apoptosis is a key determinant of necrosis in advanced plaques^5^. Using a modified *in situ* TUNEL technique (described previously in refs 17,24) we examined whether macrophage-specific loss of TRPC3 had any effect in the accumulation of apoptotic macrophages in advanced lesions. Simultaneous staining of aortic root sections for TUNEL and CD68 revealed that plaques from MacTrpc3^−/−^/Ldlr^−/−^ → Ldlr^−/−^ mice had a significantly reduced number of apoptotic macrophages (CD68^+^TUNEL^+^ cells) compared to controls [25.43 ± 5 cells/mm^2^ (n = 6) vs. 8.40 ± 1 cells/mm^2^ (n = 6), for Ldlr^−/−^ → Ldlr^−/−^ and MacTrpc3^−/−^/Ldlr^−/−^ → Ldlr^−/−^ mice, respectively, p = 0.008; Supplemental Figure S5].

**Figure 4:**
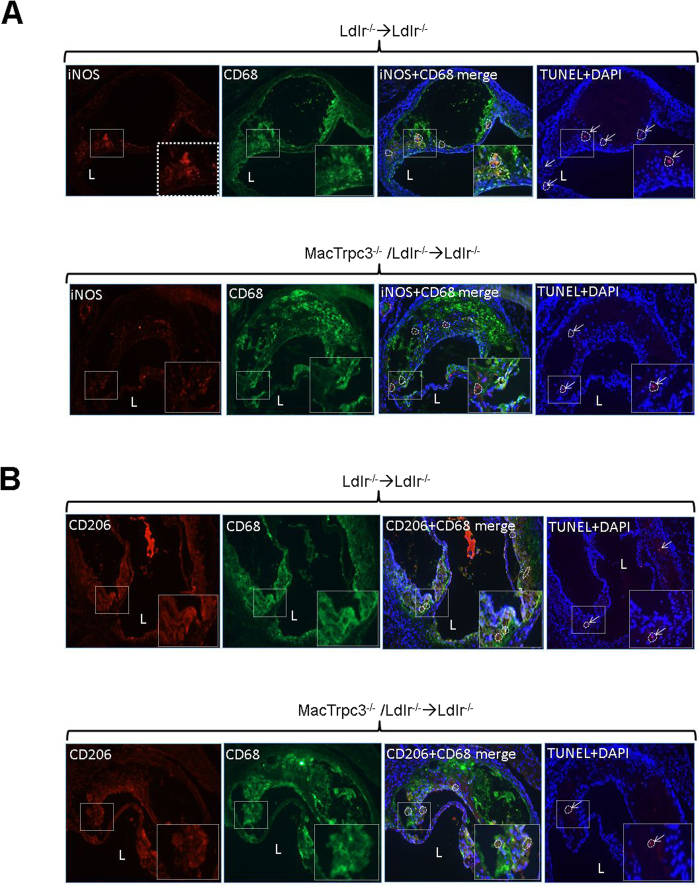
Aortic root sections from Ldlr^−/−^ → Ldlr^−/−^ or MacTrpc3^−/−^/Ldlr^−/−^ → Ldlr^−/−^ mice that were maintained on high fat diet for 14 weeks were processed for (**A**) co-immunostaining for iNOS (M1 marker, red) and CD68 (macrophage marker, green), and the corresponding consecutive section processed for *in situ* TUNEL to detect apoptotic cells. Nuclei were stained with DAPI. (**B)** co-immunostaining for mannose receptor (CD206, M2 marker, red) and CD68 (green), and the corresponding consecutive section processed for *in situ* TUNEL. The arrows point to TUNEL^+^ cells; dotted line areas delineate iNOS^+^CD68^+^ or CD206^+^CD68^+^ cells. Typical immunoreactive areas (white-dotted box in full image) are shown at higher magnification in the insets. Total magnification: 100x.

In a recent study we showed that, *in vitro*, macrophage-specific loss of TRPC3 selectively reduces the susceptibility of M1, but not that of M2 macrophages, to ER stress-induced apoptosis^18^. Therefore, to gather insight whether this was also the case for polarized macrophages in the plaque setting, we evaluated the number of TUNEL-positive cells that overlapped with iNOS^+^CD68^+^ (M1 macrophages) or with CD206^+^CD68^+^ (M2 macrophages) cells. As shown in Fig. 4, immunoreactivity for M1 and M2 macrophages was clearly noticeable in plaques from both groups of mice. Interestingly, the comparison of sections co-immunostained for M1 macrophages (iNOS^+^CD68^+^) with the corresponding TUNEL-stained consecutive sections, showed that plaques from MacTrpc3^−/−^/Ldlr^−/−^ → Ldlr^−/−^ mice had a significantly reduced number of apoptotic cells overlapping with M1 macrophages than lesions from Ldlr^−/−^ → Ldlr^−/−^ mice [respectively, 3.9 ± 0.3 (n = 12) vs. 5.8 ± 0.3 (n = 9), TUNEL^+^ cells overlapping with iNOS^+^CD68^+^ cells, per total M1 cells, p = 0.02, Fig. 4A]. Applying the same analysis we did not observe significant differences in the number of apoptotic cells overlapping with CD206^+^CD68^+^ cells (M2 macrophages) [6.13 ± 1.26 (n = 8) vs. 7.07 ± 2.53 (n = 6), TUNEL^+^ cells overlapping with CD206^+^CD68^+^ cells per total M2 cells, for MacTrpc3^−/−^/Ldlr^−/−^ → Ldlr^−/−^ vs. Ldlr^−/−^ → Ldlr^−/−^ mice, respectively, p = 0.77, Fig. 4B]. To further characterize the phenotype of iNOS^+^ or CD206^+^ cells within CD68 immunoreactive areas, we isolated iNOS^+^ or CD206^+^ cells co-localizing with CD68^+^ (macrophages) cells from aortic root plaques of Ldlr^−/−^ → Ldlr^−/−^ and MacTrpc3^−/−^/Ldlr^−/−^ → Ldlr^−/−^ mice using laser capture microdissection (LCM), and evaluated the expression level of additional M1 and M2 markers (Supplemental Figure S6). As expected, iNOS, Arg2 and TNFα were highly expressed in LCM-captured iNOS^+^ cells, compared to the M2 markers Arg1, CD206 and Ym1. The opposite pattern was observed in LCM-captured CD206^+^ cells. There were no differences in the expression level or distribution of M1 and M2 markers between macrophages isolated from Ldlr^−/−^ → Ldlr^−/−^ and MacTrpc3^−/−^/Ldlr^−/−^ → Ldlr^−/−^ mice. The almost undetectable expression of Trpc3 in LCM-captured macrophages from plaques of MacTrpc3^−/−^/Ldlr^−/−^ → Ldlr^−/−^ mice confirms deletion of macrophage Trpc3 in the lesion setting.

**Figure 5:**
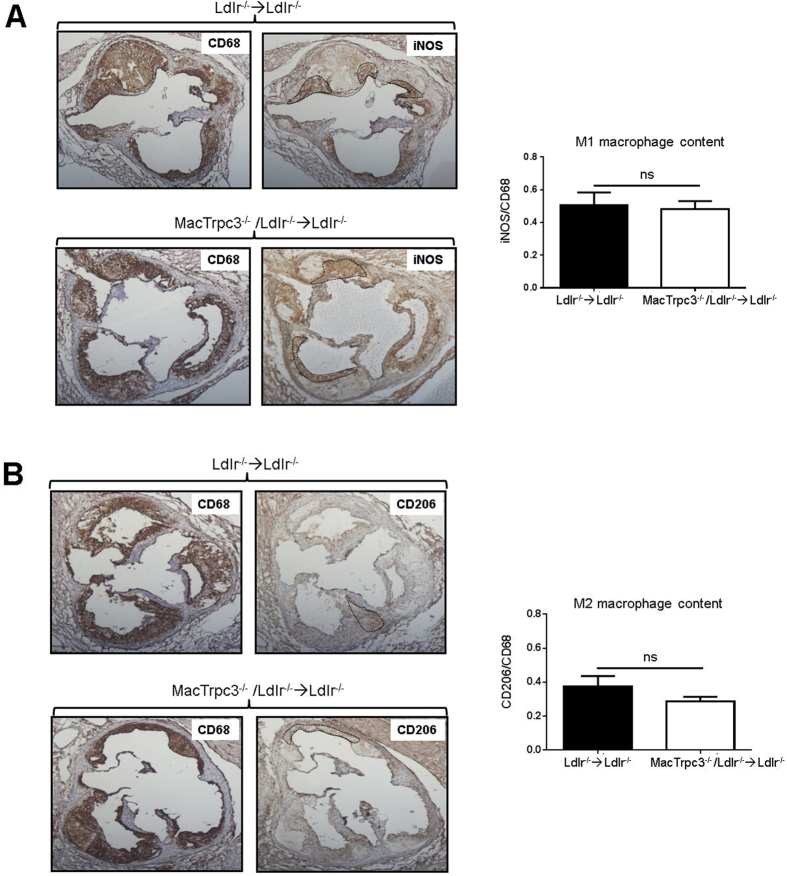
Aortic root sections from Ldlr^−/−^ → Ldlr^−/−^ or MacTrpc3^−/−^/Ldlr^−/−^ → Ldlr^−/−^ mice that were maintained on high fat diet for 14 weeks, were immunostained for (**A**) CD68 (total macrophage content) or iNOS (M1 macrophages), (**B**) CD68 or CD206 (M2 macrophages), as indicated. Stainings for CD68 and iNOS or CD68 and CD206, were performed on consecutive sections. The quantitation (mean ± SEM) of stained areas for iNOS and CD206 were normalized by total CD68-positive area; n = 5, ns: not statistically significant difference. The dotted lines show representative iNOS^+^ or CD206^+^ areas that overlap with CD68^+^ areas. Total magnification: 50X.

To examine whether the effect of macrophage-deficiency of Trpc3 on apoptosis of M1 macrophages had an impact on their relative abundance in lesions from MacTrpc3^−/−^/Ldlr^−/−^ → Ldlr^−/−^ mice compared to Ldlr^−/−^ → Ldlr^−/−^ animals, we measured the extent of iNOS and CD206 immunoreactivity overlapping with CD68-positive areas, and compared the relative areas within and between groups. As shown in Fig. 5, there were no significant differences in the proportions of M1 or M2 macrophages between MacTrpc3^−/−^/Ldlr^−/−^ → Ldlr^−/−^ and Ldlr^−/−^ → Ldlr^−/−^ mice; accordingly, the M1/M2 ratios were not different between the two groups [M1/M2 ratios: 1.43 ± 0.13 vs. 1.69 ± 0.09, for plaques from Ldlr^−/−^ → Ldlr^−/−^ and MacTrpc3^−/−^/Ldlr^−/−^ → Ldlr^−/−^ mice, respectively; n = 6, p = 0.117].

## Discussion

The present study was aimed at examining the impact of macrophage-specific loss of TRPC3 on typical features of advanced atherosclerotic lesions in a mouse model of atherosclerosis. Two major findings were made. First, the lack of macrophage TRPC3 was associated to an important reduction in plaque necrosis, compared to lesions in mice from the control group. Second, plaques in mice with macrophage-specific loss of TRPC3 had a significantly reduced number of apoptotic cells that co-localized with regions immunoreactive for M1 macrophages, in comparison to control animals. These findings are important not only in the general context of the effects of TRPC3 in macrophage biology and atherosclerosis, but also in the light of our previous *in vitro* and *in vivo* studies on the specific roles of macrophage TRPC3 in atherorelevant processes.

In previous work using Apoe knockout mice transplanted with bone marrow from mice with global deficiency of Trpc3 we also found a reduction in necrosis and in the number of apoptotic macrophages in advanced stage atherosclerotic lesions^17^. Considering that mouse lymphocytes, neutrophils and dendritic cells do not express TRPC3^19^,^20^,^21^,^22^,^23^, it was then concluded that the findings in ApoeKO mice were, presumably, the consequence of an effect of Trpc3 deficiency on plaque macrophages^17^. However, since the donor mice were global knockouts for Trpc3, this model gave rise to the question whether Trpc3 deletion in bone marrow cells other than myeloid precursors could have contributed to the observed plaque phenotype^17^. In this context, the present findings specifically address that question and provide a conclusive answer demonstrating that the loss of macrophage TRPC3 is indeed associated to a reduction in necrosis and in macrophage apoptosis in plaques.

When we first examined the effect of TRPC3 on mechanisms of macrophage survival/apoptosis, we found that the loss of TRPC3 function resulted in increased apoptosis of naïve macrophages^26^. The prediction that this effect would result in accumulation of apoptotic macrophages in advanced plaques of mice with macrophage loss of TRPC3 is proven wrong by both the present findings and the previous observations in the ApoeKO bone marrow transplantation model^17^. Indeed, from these observations it was obvious that in the plaque setting the impact of Trpc3 deficiency on apoptosis of macrophages was quite different from what we found *in vitro* in naïve macrophages. Since M1 and M2 macrophages are the predominant types in atherosclerotic lesions^27^,^28^, we concluded that the *in vivo* findings in the ApoeKO transplantation model were likely reflecting a distinctive effect of TRPC3 on polarized macrophages^17^. This interpretation was strongly supported by our recent *in vitro* studies showing that M1, but not M2 macrophages prepared from mice with macrophage-specific loss of TRPC3 have a significant reduction in their susceptibility to undergo apoptosis when subjected to chronic ER stress, a well-recognized mechanism that promotes macrophage death in the plaque setting^17^,^18^. The *in vivo* relevance of these *in vitro* observations is, to a great extent, supported by the findings in the present work. Indeed, the atherosclerotic plaques in mice with macrophage-specific loss of TRPC3 have, compared to control animals, a reduced number of apoptotic cells that co-localize with areas immunoreactive for iNOS and CD68, presumably indicative of reduced number of apoptotic M1 macrophages.

The reduction in plaque necrosis in mice with macrophage-specific loss of TRPC3 was not accompanied by changes in plaque size or lipid content, which were not different compared to control animals. This lack of correlation between changes in necrotic core size and plaque burden is not uncommon, and is thought to be related to the fact that necrosis results from a complex interaction between macrophage apoptosis, clearance of apoptotic bodies, trafficking of macrophages and/or *in situ* proliferation, all of which do not necessarily parallel plaque growth^29^.

More unexpected however, was the lack of correlation between the reduction in number of apoptotic macrophages and the plaque macrophage content in mice with macrophage-specific loss of TRPC3. Intuitively, in the advanced plaque setting where cell egress is impaired^30^,^31^, one would anticipate that reduced apoptosis should lead to accumulation of surviving cells and this, while delaying necrotic core growth, would result in increased plaque cellularity and size. Clearly, this was not the case in the MacTrpc3^−/−^/Ldlr^−/−^ → Ldlr^−/−^ mice. Of importance, efferocytosis in the plaque environment was not affected by macrophage-deficiency of Trpc3, since *in situ* efferocytosis, as assessed from the ratio of “free” vs. “macrophage-associated” apoptotic cells, was similar between plaques of Ldlr^−/−^ → Ldlr^−/−^ and MacTrpc3^−/−^/Ldlr^−/−^ → Ldlr^−/−^ mice (Supplemental Figure S7). Altogether, these findings suggest that some sort of relocation of surviving M1 cells may have occurred, which prevented their accumulation from causing changes in plaque volume. Interestingly, in a recent study aimed at analyzing the transcriptome of M1 macrophages, we found marked changes in expression levels of several genes linked to cell movement in Trpc3-deficient M1 macrophages compared to Trpc3-expressing cells, suggesting that alterations exist in motility pathways in Trpc3-deficient M1 macrophages^32^. This contention was supported by data from an *in vitro* migration assay showing that Trpc3-deficient M1 macrophages exhibit increased migratory response to CCL2^32^. Additional studies are needed to specifically examine the effect of Trpc3 deficiency on the migration capability of M1 macrophages *in vivo*.

In humans, rupture of vulnerable plaques causes an important number of acute thromboembolic events^3^,^33^. Indeed, positive correlations have been found between the size of the necrotic core and the incidence of ruptured plaques, the area of necrosis often representing more than 25% of the total plaque area^33^. By analogy, one thus would anticipate that the reduction of 40% in necrosis content observed here in mice with macrophage-specific loss of TRPC3 should be significant in the context of plaque stability. However, with few exceptions, plaque rupture is a controversial event in mice as there is no indication that fibrous cap rupture with luminal thrombosis truly occurs in these animals^34^. Also, the histomorphometric analysis in the present study was conducted in plaques of the aortic root, and there is no evidence of plaque disruptions occurring in this vascular bed site^35^,^36^,^37^. The brachiocephalic artery is a preferred site for plaque stability studies because plaque disruptions do occur and it models human pathology better than other anatomical locations in the mouse^35^,^38^. In the timeframe of our studies we did not find advanced plaques in the brachiocephalic artery, and thus longer periods of high fat feeding may be required to conduct lesion stability analysis at this vascular site. When that information becomes available, the present observations will be of value to evaluate if and to what extent changes in surrogate parameters in the aortic root correlate with similar measures in the brachiocephalic artery.

Most desirable goals in the clinical management of atherosclerosis are to reduce the progression of plaques, stimulate their regression, and/or improve stability of vulnerable plaques. Macrophages are central players in all of these processes. Yet, the potential applicability to human disease of strategies aimed at manipulating apoptosis – a major contributor to necrosis and plaque instability- or migration –key to plaque regression- of plaque macrophages is still under debate. An often disregarded aspect is that macrophages in the plaque are phenotypically and functionally heterogenous. Acknowledging the distinctive impact of macrophage diversity on plaque development and progression is important, as it may lead to definitions to design alternative therapeutic strategies. The identification of molecular elements that specifically contribute to atherorelevant functions of a particular macrophage type but not others remains an unmet goal. Filling this gap in knowledge may drive drug development towards targeting detrimental events in a phenotype selective manner, while minimally interfering with the desirable types. For instance, in mouse models of plaque regression, successful reduction in plaque burden is often paralleled by enrichment in M2 macrophages^39^,^40^. It has been shown that ER stress favors differentiation of M2 macrophages, and suppression of ER stress shifts M2s towards an M1 phenotype^41^. Intuitively, this suggests that interventions aimed at targeting components of the unfolded protein response as a strategy to reduce ER stress-induced apoptosis with no discrimination of phenotypes, may favor M2-to-M1 conversion. This would have a predictable negative effect on necrosis and/or regression of advanced plaques. Thus, manipulating apoptosis of plaque macrophages in a phenotype selective manner is an attractive, alternative strategy to potentially improve stability and/or eventually favor regression of plaques. Molecular elements that contribute to these processes in a phenotype selective manner have not been so far described. In this context, the present findings supporting a selective effect of Trpc3 on apoptosis of M1 macrophages in the plaque setting, may represent a first step towards reaching that goal.

## Materials and Methods

### Experimental animals

All animal studies described in this work conform to the Guide for Care and Use of Laboratory Animals published by the NIH and have been approved by the University of Toledo Institutional Animal Care and Use Committee. Generation and characterization of LysMCre^+/−^/Trpc3^lox/lox^ mice (for simplicity, MacTrpc3^−/−^) was described in detail in ref. 18. MacTrpc3^−/−^/Ldlr^−/−^ mice were generated by crossing MacTrpc3^−/−^ (C57BL/6 background) with low density lipoprotein receptor knockout (“Ldlr^−/−^”, B6.129S7-Ldlr^tm1Her/J^, Jackson Labs, ME) for 10 generations. All colonies were maintained in our animal facility (Division of Laboratory Animal Research, University of Toledo). Euthanasia was performed by intraperitoneal injection of sodium pentobarbital (150 mg/kg) added to an anti-coagulant (heparin, 10 Units/ml).

### Preparation of bone marrow-derived macrophages

Culture of bone marrow-derived macrophages, *in vitro* differentiation to the M1 or M2 types and phenotypic marker profiling was performed as in ref. 18.

### Bone marrow transplantation (BMT)

This was conducted essentially as we described in ref. 17. Briefly, recipient mice (Ldlr^−/−^ females, 6-week-old, C57BL/6 background) were irradiated (10 Gy, 3 min; ^137^Cs-Gammacell 40 Exactor, Nordion Int. Inc.) and 4 hours later injected via tail vein with bone marrow cells (~5 × 10^6^ cells) from Ldlr^−/−^ or MacTrpc3^−/−^/Ldlr^−/−^ mice. Preparation of bone marrow cells from donors is described in ref. 17. Conversion to the donor’s phenotype was confirmed four weeks after transplantation by PCR of genomic DNA from peripheral blood (Supplemental Figure S1). Recipients of bone marrow from Ldlr^−/−^ mice exhibited the expected 380 bp amplicon (wild-type Trpc3), whereas recipients of bone marrow from mice with macrophage-specific loss of Trpc3 showed the 461 bp amplicon corresponding to the floxed Trpc3 gene. The primers used for Trpc3 were: F: GAT GGC TCA GCA GTT AAA AGC TCT GG; R: GAA GTC ACT TCA GAC AGT CCA AAT AT. PCR for LDLR was performed using the primers and conditions recommended by Jackson Labs (Jackson Labs, ME).

### Determination of plasma cholesterol and triglycerides

After 12 h of fasting blood was collected by submandibular vein puncture. Total plasma cholesterol and triglycerides were determined using Cholesterol-E and L-Type Triglyceride-M (Wako Chemicals USA, Inc.) following manufacturer’s instructions.

### Aortic root sectioning

Aortic root sections were prepared as described in ref. 42. Briefly, euthanized mice were perfused through the left ventricle with 4% paraformaldehyde followed by PBS. The heart was cut so that all three aortic valves were in the same geometric plane. The upper portion of the heart was embedded in O.C.T., frozen in the Peltier stage of the cryostat (Thermo Scientific R. Allan HM550 Cryostat) and processed for sectioning. Sections (10 μm) were collected onto Fisher Superfrost Plus-coated slides, starting from where aorta exits the ventricle and moving towards the aortic sinus over ~650–700 μm. Sections were collected following the scheme that we used in ref. 42, which is based on that described by Daugherty and Whitman^43^. Essentially, each slide (at least 5 slides per mouse) contained 9–12 aortic root sections collected at 40 μm intervals; with this scheme, consecutive, or immediately adjacent sections –which are morphologically and compositionally identical- are located in separate slides, allowing for different stainings to be conducted. Additional sections were collected at the end to be used as controls in immunostaining procedures. Lesion analysis, Oil Red O (ORO) and hematoxylin and eosin (H&E) stainings were as described in ref. 17. Areas of necrosis and cap thickness were evaluated as we describe in detail in ref. 17. With this protocol, cap thickness is assessed from the largest necrotic cores from at least duplicate sections and measuring the thinnest section of the cap as determined by the distance between the outer edge of the cap and the necrotic core border. Buried caps in deep necrosis areas, when present, were not considered for measurements. Collagen content was evaluated in sections stained with Movat’s pentachrome (American MasterTech, CA) following the manufacturer’s instructions. Staining for *in situ* TUNEL was performed using an *in situ* cell death detection kit (Roche, IN) as in refs 44,45, with the modifications described in ref. 17.

### Immunohistochemistry

Immunohistochemistry (IHC) was performed using the Vecstatin Elite ABC kit (Vector Labs) and following the manufacturer’s instructions. Briefly, sections were fixed in acetone, endogenous peroxidase quenched in 0.3% hydrogen peroxide in methanol, and processed for immunostaining for CD68 (#MCA1957GA, Bio-Rad), inducible nitric oxide synthase (“iNOS”, antibody #ab15323, Abcam), mannose receptor (“CD206”, antibody #ab64693, Abcam) or α-smooth muscle actin (“αSMA”, antibody #ab5294, Abcam). After treatment with secondary antibodies (for CD68: biotinylated goat anti-rat antibody #BA-9401, Vector laboratories; for iNOS, CD206 and αSMA: biotinylated goat anti-rabbit antibody, Vecstatin Elite ABC kit, Vector laboratories), sections were incubated with Vectastain ABC reagent and peroxidase substrate solution (# SK-4100, Vector laboratories). Counterstaining was with hematoxylin. Negative controls were performed by substituting the primary antibody with non-immune IgG from the same species and at the same concentration. Under these conditions, nonspecific immunostaining was not detected. Stained areas were captured (Micropublisher 3.3 Megapixel Cooled CCD Color Digital Camera) and measured (NIS Elements D).

### Immunostaining for M1 and M2 macrophages

This was performed as in ref. 17, with modifications. M1 and M2 macrophages in aortic root sections were identified by co-staining for iNOS (#ab15323, Abcam) or mannose receptor (“CD206”, #64693, Abcam), respectively, and macrophage (CD68, #MCA1957GA, Bio-Rad), as follows: frozen sections were fixed in acetone (15 min, 4 °C), non-specific sites blocked, and this was followed by simultaneous incubation with antibodies against iNOS (1:100 dilution) and CD68 (1:300 dilution), or CD206 (1:100 dilution) and CD68 (1:300) for 3 hours. Sections were then incubated simultaneously with anti-rabbit IgG Alexa Fluor-555 (#4413 S, Cell Signaling) and anti-rat IgG Alexa Fluor-488 (#4416 S, Cell Signaling), both at 1:1,000 dilution (1 hour). Slides were mounted using the Prolong Gold Antifade Reagent with DAPI (Cell Signaling, #8961). All antibody dilutions were done using the IHC TEK antibody diluent (IW-1,000, IHC World).

### *In situ* efferocytosis

Performed as described in refs 46,47, by simultaneously staining sections for TUNEL, CD68 and nuclei (DAPI). *In situ* efferocytosis was estimated by counting number of free vs. macrophage-associated apoptotic cells within plaques. “Free” apoptotic bodies were defined as TUNEL^+^ nuclei neither surrounded nor in contact with macrophages (CD68^+^ cells). TUNEL^+^ cells overlapping or in juxtaposition to macrophages were considered “macrophage-associated”. “Free” vs. “macrophage-associated” apoptotic bodies were evaluated by 2 independent operators blinded to the samples (concurrence between the operators was >90%).

### Laser capture microdissection of plaque macrophages

iNOS^+^ or CD206^+^ cells within CD68^+^ immunoreactive areas in plaques from aortic root sections (6 μM) were isolated by laser capture microdissection (LCM) essentially as described in ref. 48. Total RNA (3 pools of RNA per group, each from 2 mice) was prepared and used to measure the expression level of Trpc3, M1 markers (iNOS, Arg2, TNFα) and M2 markers (Arg1, CD206, Ym1) by qRT-PCR. The primers used were described in refs 17,18. To estimate the enrichment in macrophage-derived RNA over whole-section RNA, we examined the expression levels of the macrophage-specific marker *Cd68* and the smooth muscle cell marker α-smooth muscle actin (*αSma*) in LCM-captured iNOS^+^ and CD206^+^ cells. The LCM-derived RNA was markedly enriched (~30 fold) in mRNA for CD68 compared to whole-section RNA, whereas that of α-Sma was negligible; cyclophilin-A was used as control gene. LCM was performed on 6–8 aortic root sections per mouse using an Arcturus PixCell II LCM Instrument, following the protocol described in ref. 48. Approximately 300 laser pulses/section (15 μm laser diameter, 40 mW power, 3 ms) were captured, RNA extracted (Arcturus Picopure RNA kit), amplified, purified and used for qRT-PCR.

### Quantitative Real-time PCR (qRT-PCR)

Was performed essentially as in ref. 18. Briefly, total RNA was prepared from bone marrow-derived macrophages using PerfectPure RNA Tissue kit (5Prime, MD) according to manufacturer’s instructions. cDNA was synthesized with oligo dT primers and reverse transcriptase (Applied Biosystems high capacity cDNA RT kit #4368814) using 1 μg of total RNA. cDNA was used for semi-quantitative Real-time PCR (qRT-PCR) using TrueAmp SYBR green qPCR supermix (Applied Biosystems, CA). The relative amount of mRNA was normalized relative to glyceraldehyde 3-phosphate dehydrogenase (Gapdh). The primers used in qRT-PCR are described in ref. 18.

### Statistical analysis

Values are shown as mean ± SEM and corresponding “n” numbers indicated in figure legends or text. Statistical differences were determined using the Student’s-t-Test with Prism Graph Pad version 6 for Windows 2007 (Graph Pad Software, San Diego CA). P values of less than 0.05 were considered significant.

## Additional Information

**How to cite this article:** Solanki, S. *et al*. Reduced Necrosis and Content of Apoptotic M1 Macrophages in Advanced Atherosclerotic Plaques of Mice With Macrophage-Specific Loss of Trpc3. *Sci. Rep.* **7**, 42526; doi: 10.1038/srep42526 (2017).

**Publisher's note:** Springer Nature remains neutral with regard to jurisdictional claims in published maps and institutional affiliations.

## Supplementary Material

Supplementary Information
