# Supplementary material for: Reduced Necrosis and Content of Apoptotic M1 Macrophages in Advanced Atherosclerotic Plaques of Mice With Macrophage-Specific Loss of Trpc3
Source: Sci Rep. 2017 Feb 10;7:42526. doi: 10.1038/srep42526 (PMC5301208; doi:10.1038/srep42526)

Supplementary information

REDUCED NECROSIS AND CONTENT OF APOPTOTIC M1 MACROPHAGES IN ADVANCED ATHEROSCLEROTIC PLAQUES OF MICE WITH MACROPHAGE-SPECIFIC LOSS OF TRPC3

Sumeet Solanki, Prabhatchandra R. Dube, Lutz Birnbaumer and Guillermo Vazquez

Table S1.

Body weight and lipid profile for $Ldlr^{-/-}$→$Ldlr^{-/-}$ and $MacTrpc3^{-/-}$/$Ldlr^{-/-}$→$Ldlr^{-/-}$ mice. Mice were fed a high fat diet for 14 weeks. Body weight and total plasma cholesterol and triglycerides were measured as described in Methods. Values are mean ± SEM, n= 11-12.

	**$Ldlr^{-/-}$→$Ldlr^{-/-}$**	**$MacTrpc3^{-/-}$/$Ldlr^{-/-}$→$Ldlr^{-/-}$**
Total cholesterol (mg/dl)	1,012 ± 87	1,089 ± 91
Triglycerides (mg/dl)	112 ± 20	123 ± 17
Body weight before diet (g)<br>Body weight after diet (g)	16.21 ± 0.17<br>21.39 ± 1.34	16.48 ± 0.25<br>18.98 ± 0.50

LEGENDS TO SUPPLEMENTAL FIGURES

Supplemental Figure S1.

Lethally irradiated female Ldlr-/- mice were transplanted with bone marrow from MacTrpc3$^{-/-}$/Ldlr$^{-/-}$ (MacTrpc3$^{-/-}$/Ldlr$^{-/-}$$\rightarrow$Ldlr$^{-/-}$, n=12) or Ldlr$^{-/-}$ mice (Ldlr$^{-/-}$$\rightarrow$Ldlr$^{-/-}$, n=12). The genotyping was performed using gDNA from peripheral blood (submandibular vein
6 puncture) collected at four weeks after bone marrow transplantation to examine Trpc3 by PCR. The 461 bp band corresponds to Trpc3 with floxed exon 7 (see 45), whereas the 380 bp band corresponds to wild-type Trpc3.

Supplemental Figure S2.

Quantitative real-time PCR on cDNA from BMDMs isolated from Ldlr$^{-/-}$$\rightarrow$Ldlr$^{-/-}$ (Trpc3$^{+/+}$BM) or MacTrpc3$^{-/-}$/Ldlr$^{-/-}$$\rightarrow$Ldlr$^{-/-}$ mice (Trpc3$^{-/-}$BM) 14 weeks after bone marrow transplantation. Data is shown as mean ± SEM (n=3). The primers used for Trpc3 were described in 18.

Supplemental Figure S3.

Aortic root sections from Ldlr$^{-/-}$$\rightarrow$Ldlr$^{-/-}$ or MacTrpc3$^{-/-}$/Ldlr$^{-/-}$$\rightarrow$Ldlr$^{-/-}$ mice that were maintained on a high fat diet for 14 weeks were stained with Oil-Red-O to evaluate neutral lipid content. The quantitation (mean ± SEM) of stained areas is shown. Ldlr$^{-/-}$ $\rightarrow$Ldlr$^{-/-}$ (n=10), MacTrpc3$^{-/-}$/Ldlr$^{-/-}$$\rightarrow$Ldlr$^{-/-}$ (n=11). Total magnification: 50x.

Supplemental Figure S4.

Aortic root sections from $Ldlr^{-/-}$→$Ldlr^{-/-}$ or $MacTrpc3^{-/-}/Ldlr^{-/-}$→$Ldlr^{-/-}$ mice maintained on a 14 week high fat diet were stained with **A)** Movat's pentachrome to evaluate collagen content (yellow-to-yellow orange staining); or **B)** immunostained for α-smooth muscle actin (αSMA) and counterstained with H&E. In both "A" and "B" representative sections and the corresponding quantitations of mean stained areas (mean ± SD) for $Ldlr^{-/-}$→$Ldlr^{-/-}$ (n=6-9) and $MacTrpc3^{-/-}/Ldlr^{-/-}$→$Ldlr^{-/-}$ (n=6-8) are shown. Total magnification: 50x.

Supplemental Figure S5.

Aortic root sections from $Ldlr^{-/-}$→$Ldlr^{-/-}$ or $MacTrpc3^{-/-}/Ldlr^{-/-}$→$Ldlr^{-/-}$ mice that were maintained on a high fat diet for 14 weeks were processed simultaneously for *in situ* TUNEL, to detect apoptotic cells (red), immunodetection of CD68 (macrophage marker, green) and nuclei staining (DAPI). Total magnification: 100x. Typical areas containing $TUNEL^{+}$ and $CD68^{+}$ cells (white dotted box in full image) are shown at higher magnification in the insets. Quantitations are shown in the bar graph (mean ± SEM, n=6).

Supplemental Figure S6.

Laser capture microdissection was applied to aortic root sections from $Ldlr^{-/-}$→$Ldlr^{-/-}$ or $MacTrpc3^{-/-}/Ldlr^{-/-}$→$Ldlr^{-/-}$ mice that were kept on high fat diet for 14 weeks, to isolate $iNOS^{+}$ or $CD206^{+}$ cells that localized within $CD68^{+}$ immunoreactive areas [$iNOS^{+}$($CD68^{+}$) and $CD206^{+}$($CD68^{+}$), respectively]. Total RNA (3 pools of RNA per group, each from 2 mice) was prepared and used to measure expression of Trpc3, M1

markers (iNOS, Arg2, TNFα) and M2 markers (Arg1, CD206, Ym1) by qRT-PCR. Differences between expression of M1 and M2 markers within the $iNOS^{+}(CD68^{+})$ group or within the $CD206^{+}(CD68^{+})$ group, and those between M1 or M2 markers in $iNOS^{+}(CD68^{+})$ vs. $CD206^{+}(CD68^{+})$, had all p values <0.0001.

Supplemental Figure S7.

Aortic root sections from $Ldlr^{-/-}$→$Ldlr^{-/-}$ or $MacTrpc3^{-/-}/Ldlr^{-/-}$→$Ldlr^{-/-}$ mice that were maintained on a high fat diet for 14 weeks were processed simultaneously for *in situ* TUNEL (apoptotic cells, red), immunodetection of CD68 (macrophage marker, green) and nuclei staining (DAPI, blue). Total magnification: 400x. “Free” and “macrophage associated” (“Mϕ-associated”) apoptotic cells –see text for definitions- were counted to determine the ratio of free:Mϕ-associated apoptotic cells as an evaluation of in situ efferocytosis. The bar graph shows mean ± SD, n=6; ns: not statistically significant difference.

Supplemental figure S1

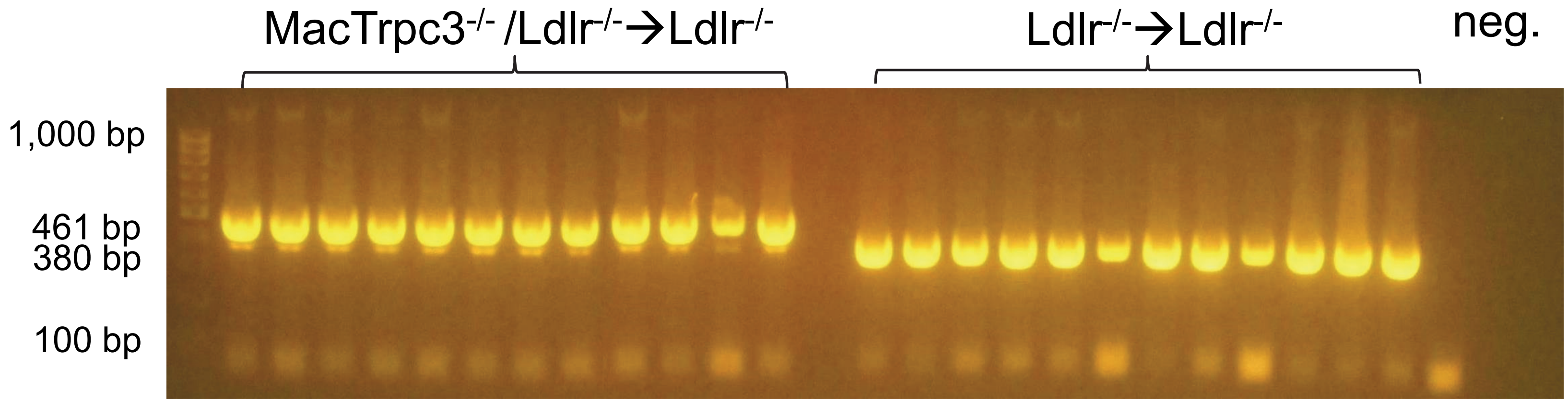

Supplemental figure S2

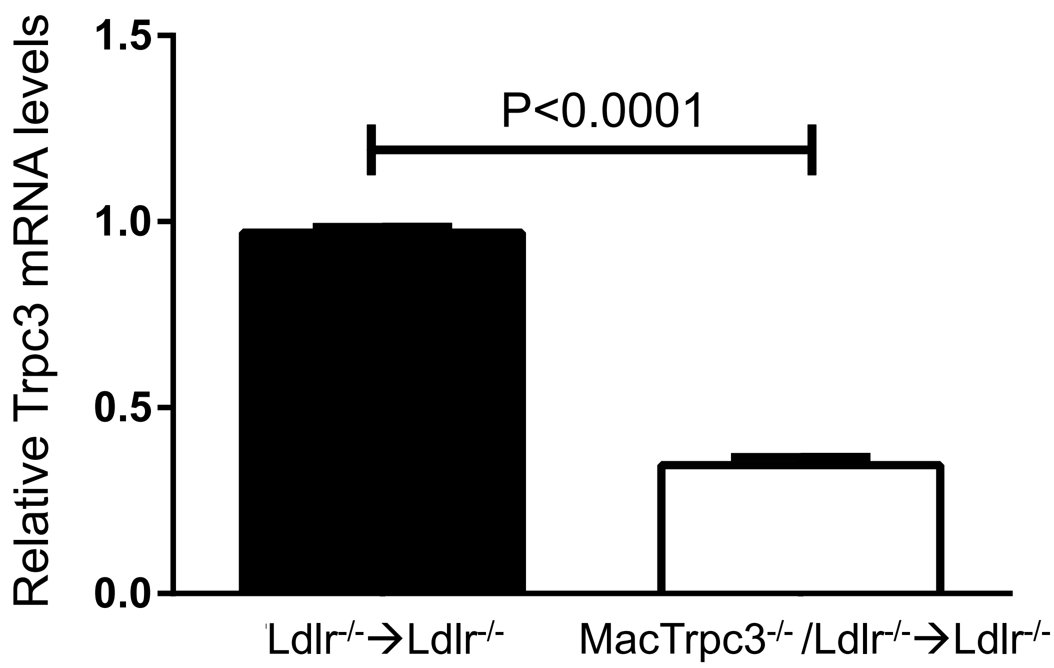

Supplemental figure S3

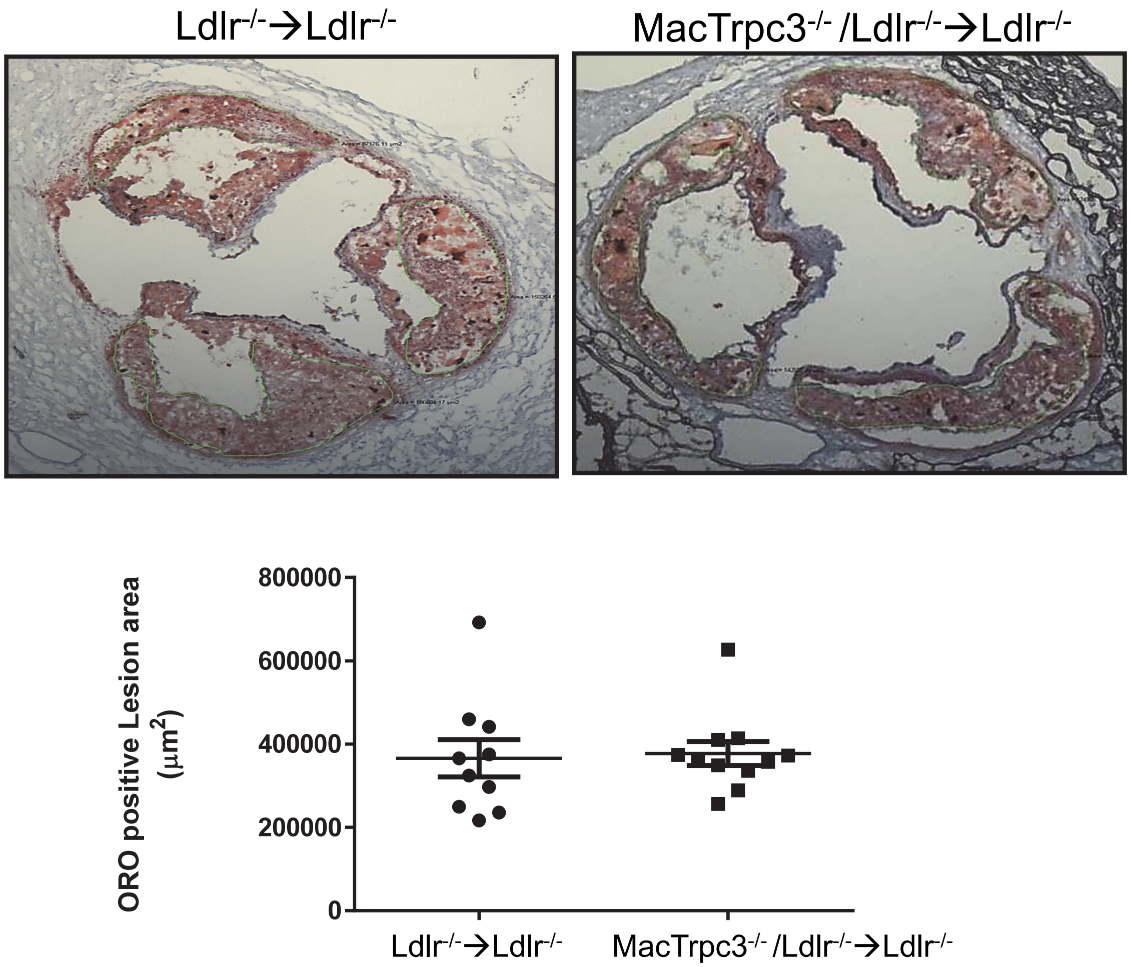

Supplemental Figure S4

A

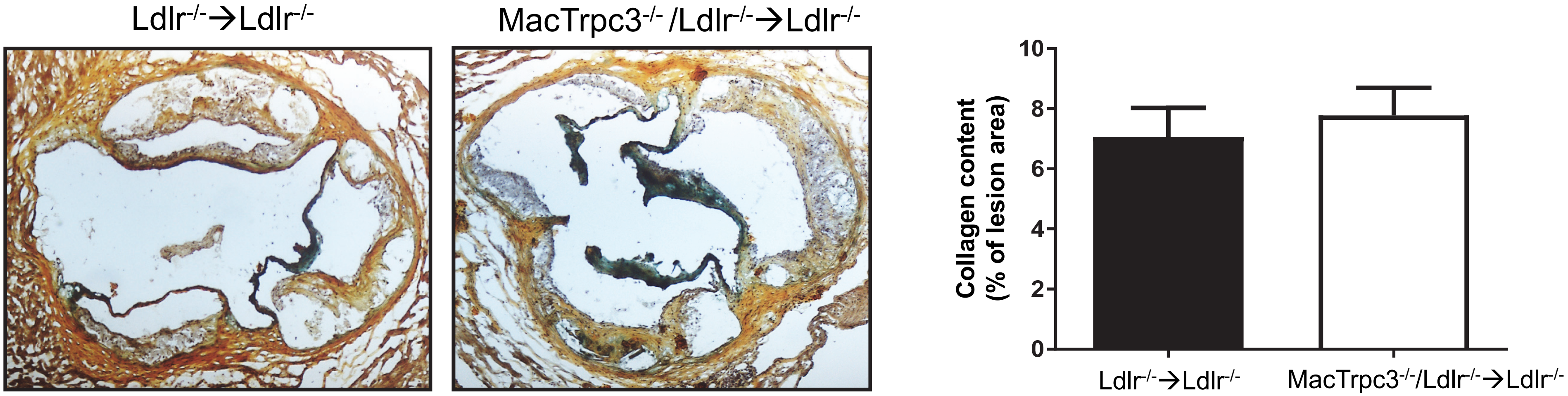

B

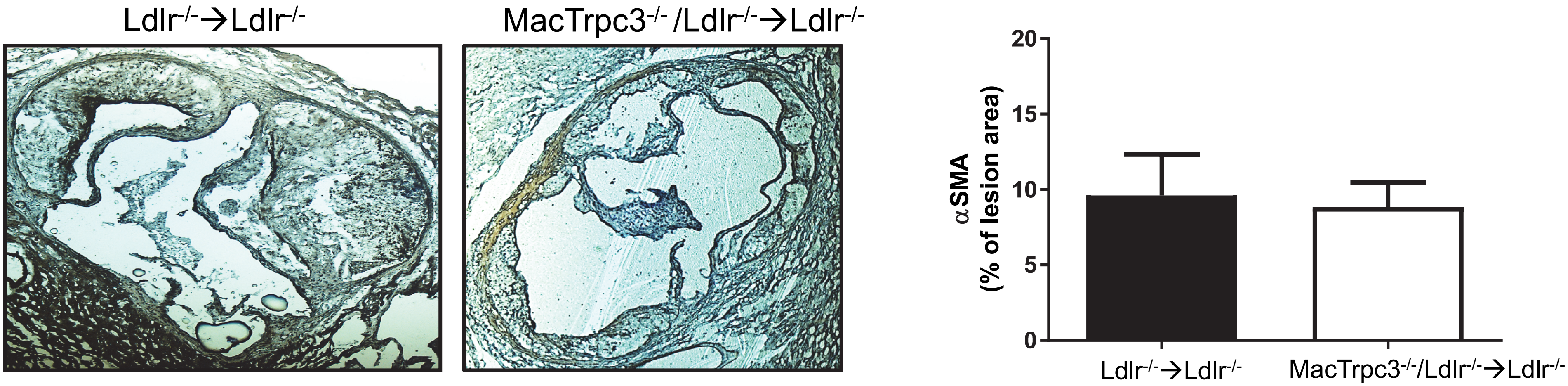

Supplemental Figure S5

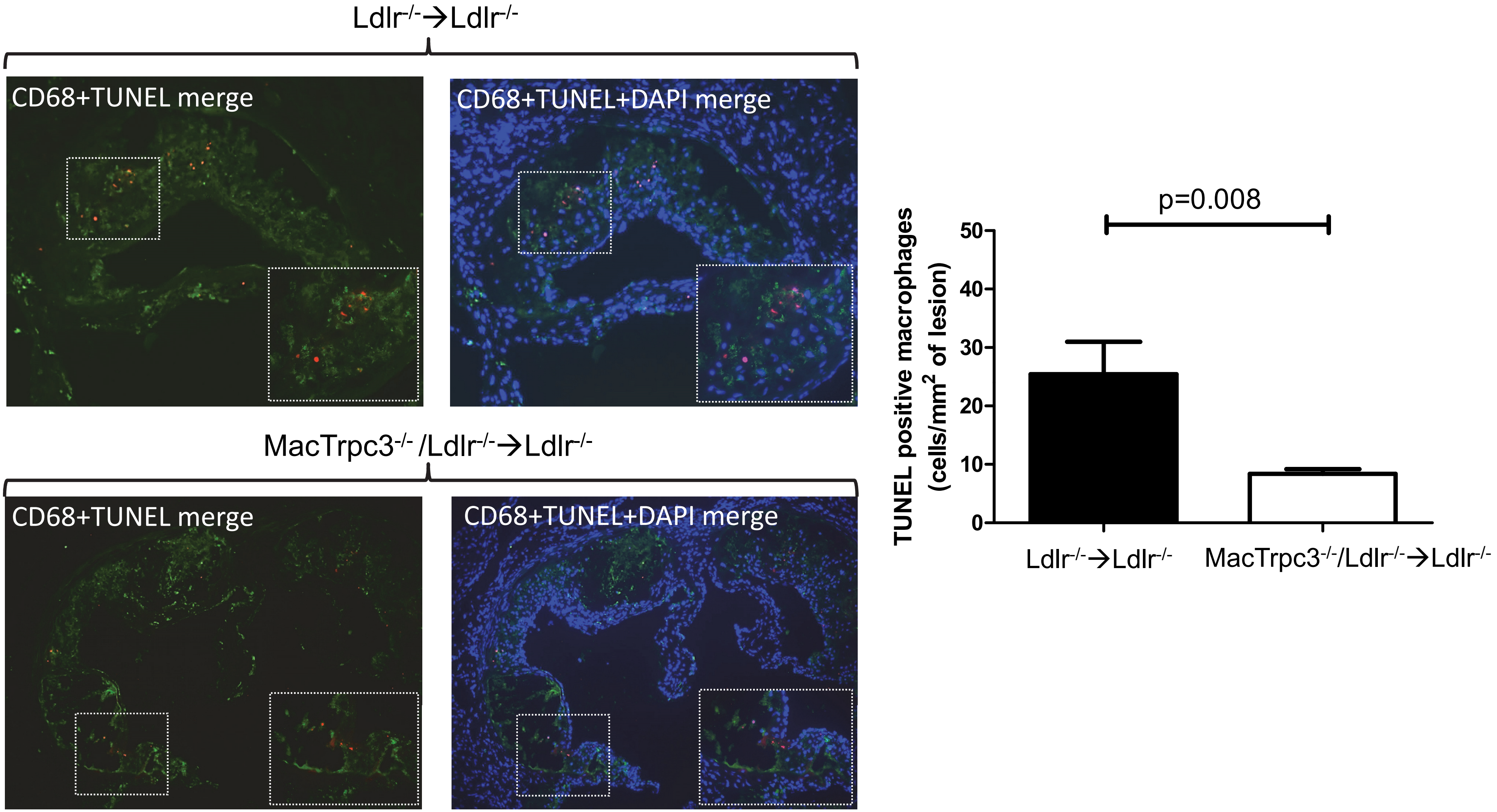

Supplemental Figure S6

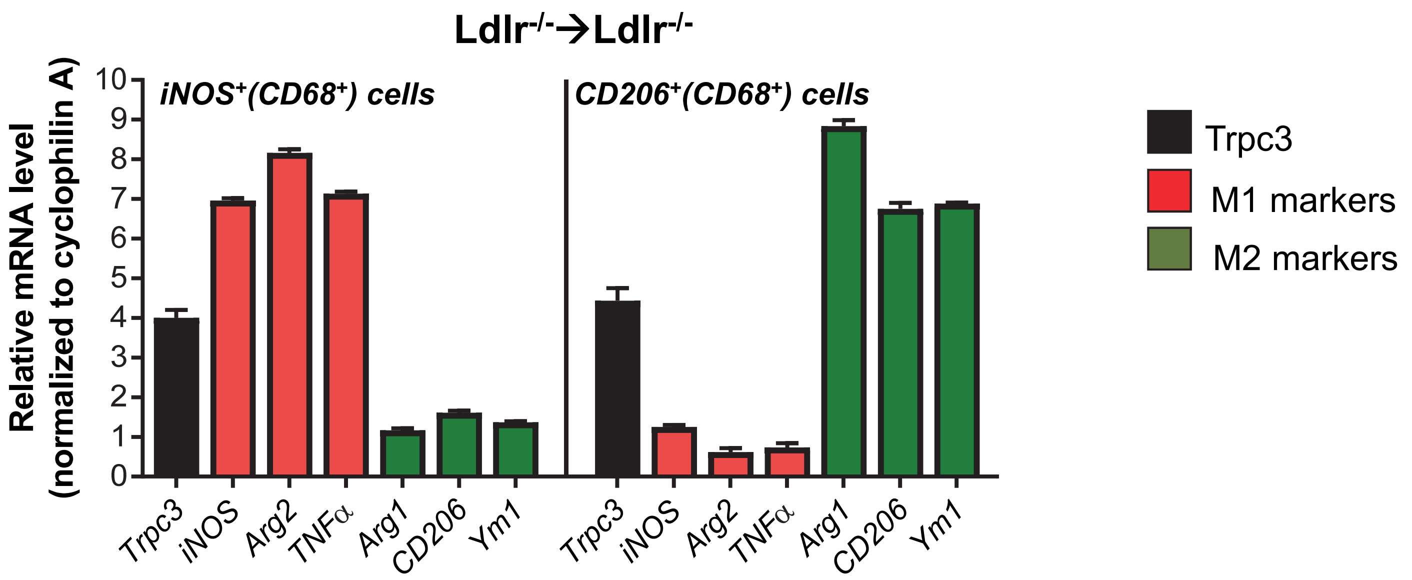

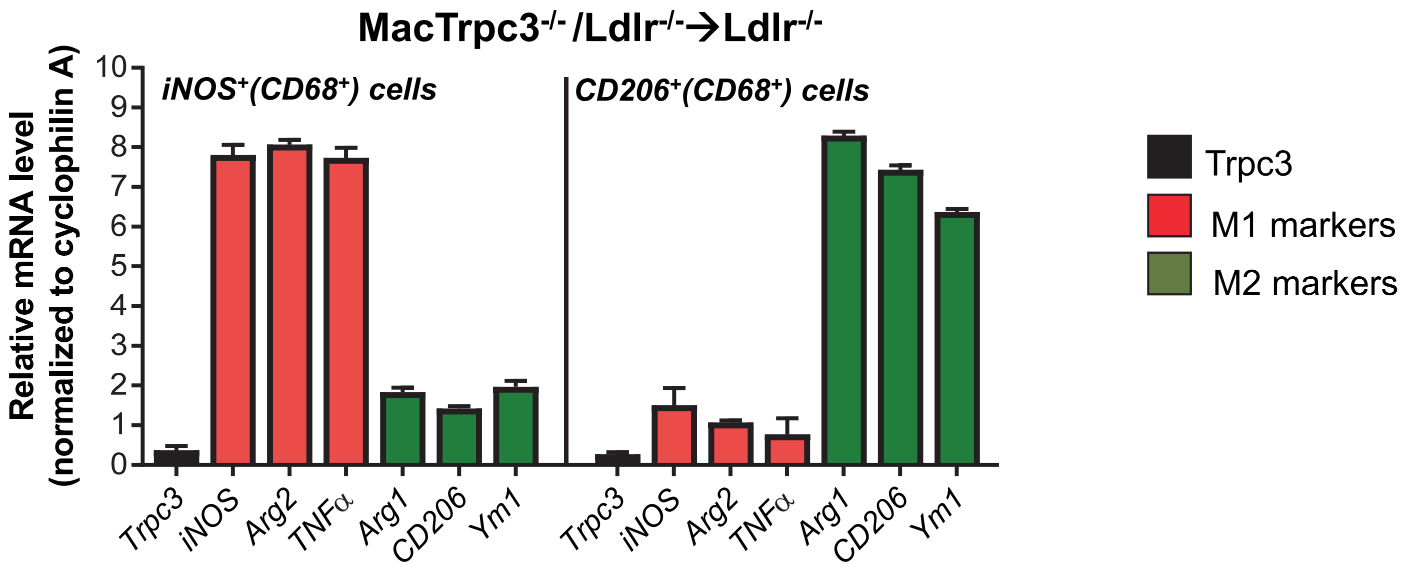

Supplemental Figure S7

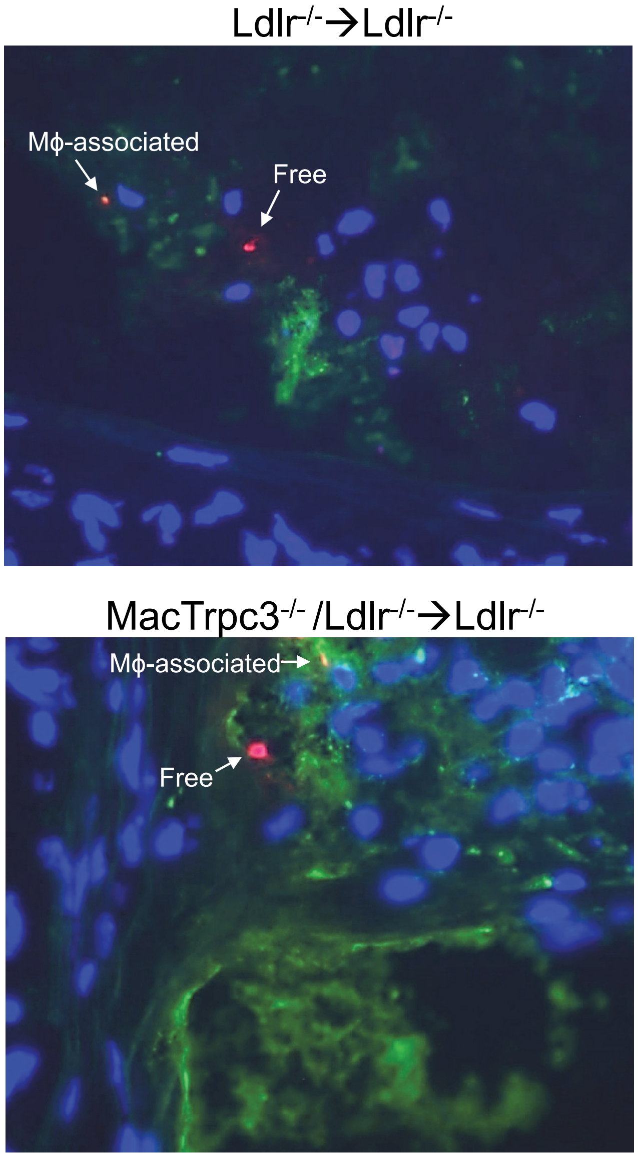

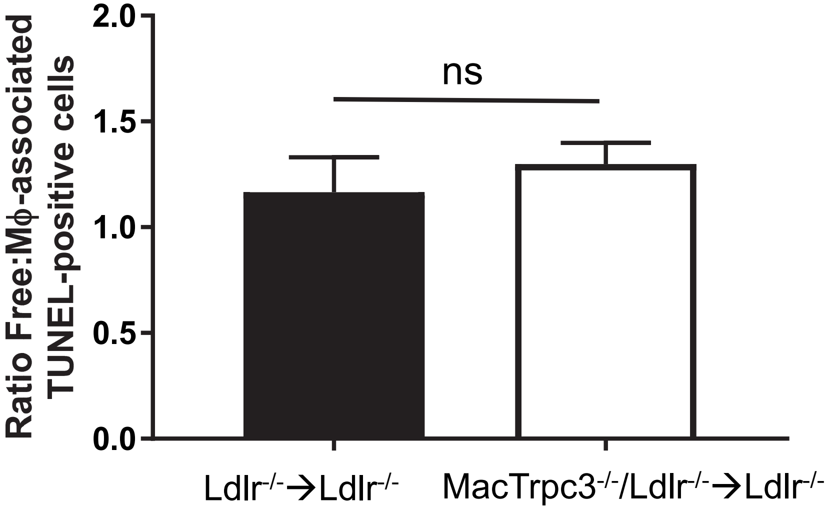

Supplement: Supplementary Information [file srep42526-s1.pdf]
